# A vegan diet improves insulin resistance in individuals with obesity: a systematic review and meta-analysis

**DOI:** 10.1186/s13098-022-00879-w

**Published:** 2022-08-13

**Authors:** Peng Chen, Ying Zhao, Yan Chen

**Affiliations:** 1 Department of Pediatrics, The Second Hospital of Jilin University, Changchun, China; 2 Department of Endocrinology, The Second Hospital of Jilin University, No. 218, Ziqiang Street, Nanguan District, Changchun, China

**Keywords:** Obesity, Insulin resistance, Type 2 diabetes mellitus, Vegan diet, Meta-analysis

## Abstract

**Background:**

A vegan diet has benefits on weight reduction and on the parameters of glucose and lipid metabolism. This meta-analysis aimed to investigate the efficacy of plant-based diets on insulin resistance and blood lipids in patients with obesity.

**Methods:**

PubMed, Embase, and the Cochrane Library were searched for available papers published up to March 2021. The primary outcome was insulin resistance which was assessed by Homeostasis Model Assessment Insulin Resistance (HOMA-IR), other metabolic parameters measures including the pre/post-diet changes in triglycerides, HDL-cholesterol, total cholesterol, LDL-cholesterol. All analyses were performed using the random-effects model.

**Results:**

Six studies (seven datasets) were included. Compared with baseline, the plant-based diet improved the HOMA-IR (SMD = 1.64, 95%CI 0.95, 2.33; I^2^ = 91.8%, P_heterogeneity_ < 0.001), total cholesterol (SMD = 2.51, 95% CI 0.88, 4.13; I^2^ = 98.0%, P_heterogeneity_ < 0.001), HDL-cholesterol (SMD = 1.55, 95% CI 0.66, 2.44; I^2^ = 92.0%, P_heterogeneity_ < 0.001), and LDL-cholesterol (SMD = 2.50, 95% CI 1.30, 3.70; I^2^ = 94.4%, P_heterogeneity_ < 0.001), but not the triglycerides (SMD = − 0.62, 95% CI − 1.92, 0.68; I^2^ = 97.8%, P_heterogeneity_ < 0.001). The sensitivity analyses showed that the results were robust.

**Conclusions:**

In obese individuals with insulin resistance, a vegan diet improves insulin resistance and dyslipidemia, except for triglycerides.

**Supplementary Information:**

The online version contains supplementary material available at 10.1186/s13098-022-00879-w.

## Background

Obesity is associated with increased morbidity and mortality, including increased risk of cardiovascular events and increased risk of certain cancers [1–4]. An estimated 12% of the world population was obese in 2015 [5]. Once a body mass index (BMI) of 25 kg/m^2^ is reached, any additional increase is associated with an increased risk of all-cause mortality [6]. Conditions such as (but not limited to) type 2 diabetes mellitus (T2DM) [7], hypertriglyceridemia [8–10], nonalcoholic fatty liver disease [11–13], and hypertension [14–16] are associated with overweight. Because each of these conditions is independently associated with increased cardiovascular risk and mortality [7–14, 17], managing body weight has a profound impact on health.

A positive energy balance (increased energy intake and/or decreased energy expenditure in relation to each other) sustained over time will lead to an increase in weight [1]. Even a 5%-15% weight loss may greatly reduce complications in persons with overweight or obesity [1]. Studies have shown that excess weight and its associated comorbidities can be favorably modified through lifestyle changes such as adopting a healthy diet and increasing energy expenditure [3, 18–21]. Nevertheless, although marked improvements have been made in initial and long-term weight losses, researchers need to identify more effective strategies to improve long-term maintenance [22].

A vegan diet is a diet that excludes animal products [23, 24], often resulting in hypocaloric diets compared with their meat-containing counterparts. By avoiding meat, vegan diets are often hypocaloric [24]. It has been suggested that persons who follow a vegan diet are more satisfied and are more likely to follow it for a longer period than other weight-loss eating plans [25–27]. Randomized controlled trials (RCTs) [28–30] and a meta-analysis [31] showed the metabolic and weight-control benefits of vegetarian and vegan diets. Consequently, a vegetarian diet has been associated with a lower risk of T2DM [32]. Indeed, the Homeostatic Model Assessment of Insulin Resistance (HOMA-IR) is an index for assessing β-cell function and insulin resistance [33], and vegetarian and vegan diets have been shown to improve the HOMA-IR [34–36]. Furthermore, since non-vegetarian diets are often rich in lipids, two meta-analyses revealed that vegetarian and vegan diets significantly decrease total cholesterol, low-density lipoprotein cholesterol (LDL-C), high-density lipoprotein cholesterol (HDL-C), and non-HDL-C, but without significant changes in triglycerides [37, 38].

This systematic review and meta-analysis aimed to investigate the efficacy of plant-based diets on the metabolic parameters of patients with obesity and insulin resistance. The results could support the use of such a diet for the management of obesity and T2DM.

## Methods

### Literature search

This systematic review and meta-analysis was conducted according to the Preferred Reporting Items for Systematic Reviews and Meta-Analyses (PRISMA) guidelines [39]. The search strategy was built using the PICOS principle [40]. PubMed, Embase, and the Cochrane Library were searched for available papers published up to March 2021 using the MeSH terms “Vegan diet”, “Insulin resistance”, and “Overweight”, as well as relevant key words, followed by screening based on the inclusion and exclusion criteria. The exact search processes are shown in Additional file 1: Table S1. For multiple articles reporting the same study population, only the most recent one and meeting the eligibility criteria were included. For articles reporting different study populations, each dataset was considered independently in this meta-analysis.

### Eligibility criteria

The eligibility criteria were (1) intervention: plant-based diet, (2) comparison: any non-vegetarian/non-vegan diet (i.e., no diet changes, Mediterranean diet, animal protein, low-fat omnivorous diet, omnivorous, beef/pork, etc.), (3) population: obese/overweight adults, (4) primary outcome: insulin resistance was assessed by Homeostasis Model Assessment Insulin Resistance (HOMA-IR) [41]; other metabolic parameters including triglycerides, HDL-cholesterol, total cholesterol, LDL-cholesterol, and (5) study design: RCT. Reviews, meta-analyses, letters to the editors, commentaries, and case reports were excluded.

### Data extraction

Study characteristics (authors, year of publication, country, and study design), patient characteristics (sex, sample size, weight, and BMI), and outcomes measured at baseline and the last assessment in the intervention group (HOMA-IR, triglycerides, HDL-cholesterol, LDL-cholesterol, and total cholesterol) were extracted and reviewed by two different investigators (P.C. and Y.C.) according to a pre-specified protocol. Discrepancies were solved by discussion until a consensus was reached.

### Quality of the evidence

The level of evidence of all articles was assessed independently by two authors (P.C. and Y.C.) according to Version 2 of the Cochrane risk-of-bias assessment tool for randomized trials (RoB 2) [42, 43]. Discrepancies in the assessment were resolved through discussion until a consensus was reached.

### Statistical analysis

All analyses were performed using STATA SE 14.0 (StataCorp, College Station, Texas, USA). The standardized mean difference (SMD) and 95% confidence intervals (CI) were used to assess the continuous variables. Statistical heterogeneity among studies was calculated using Cochran’s Q-test and the I^2^ index. An I^2^ > 50% and Q-test P < 0.10 indicated high heterogeneity. All analyses were performed using the random-effects model. P-values < 0.05 were considered statistically significant.

## Results

### Study selection

**Fig. 1:**
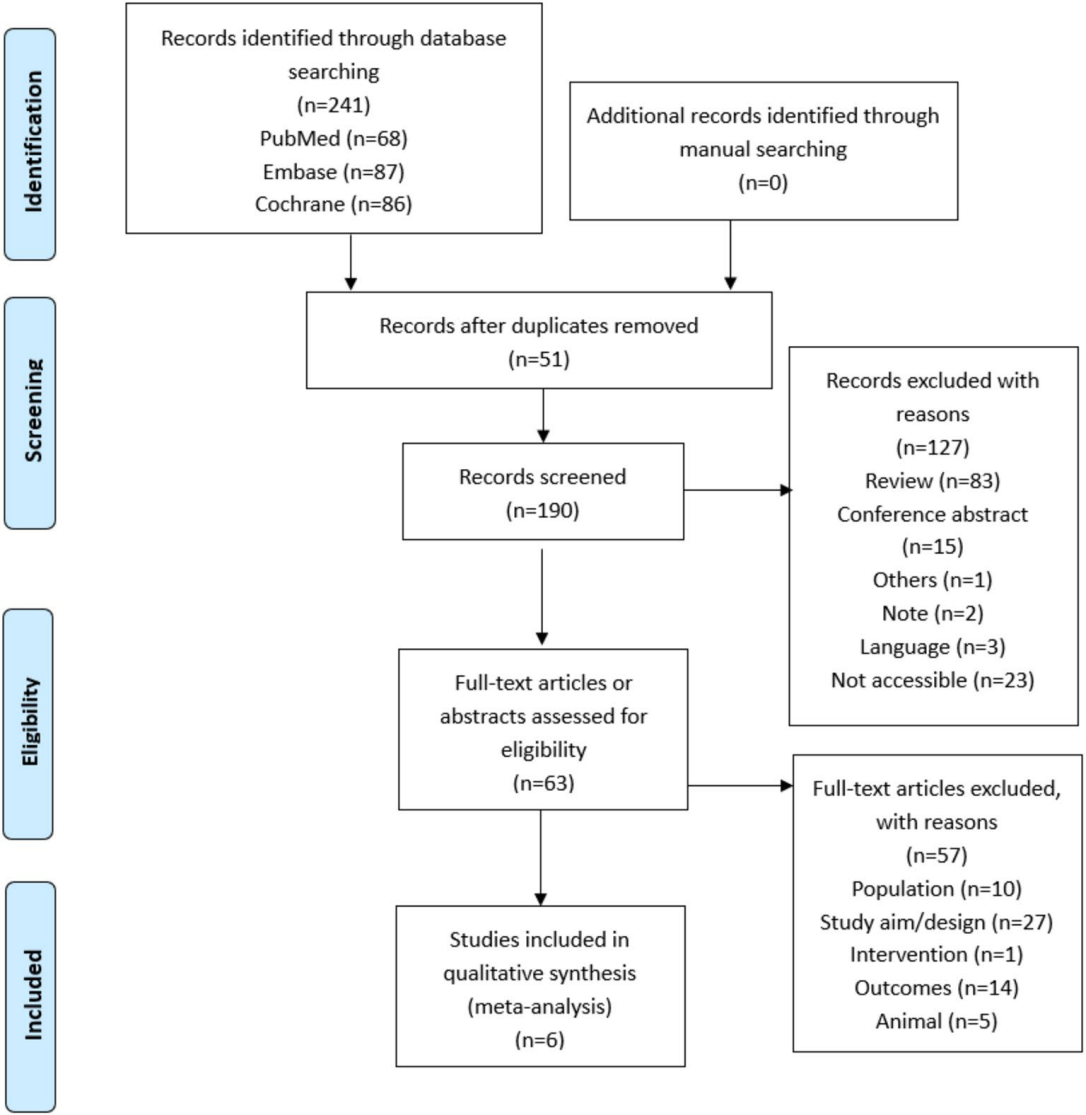
Flow diagram of the literature search and filtering results for a systematic review of the effectiveness of a plant diet on obesity

Figure 1 presents the study selection process. The initial search yielded 241 records. After removing the duplicates, 190 records were screened, and 127 were excluded (review, n = 83; conference abstract, n = 15; note, n = 1; language, n = 3; not accessible, n = 23; others, n = 1). Then, 63 abstracts or full-text papers were assessed for eligibility, and 57 were excluded (population, n = 10; study aim/design, n = 27; intervention, n = 1; outcomes, n = 14; animal study, n = 5).

Finally, six articles (seven datasets) and 303 participants were included [44–49] (Table 1). There were six RCTs [44, 45, 47–49] and one pilot study [46]. One study was from Italy [46], and five were from the United States of America [44, 45, 47–49]. The mean participants’ age varied from 44.4 to 58.3 years. The proportion of males varied from 5.3% to 91.1%. The mean participants’ BMI varied from 30.7 to 36.1 kg/m^2^. One study had a high risk of bias for two items of RoB2 [49], while the remaining studies all had an unclear risk of bias for at least one item [44–48].

**Table 1:** Characteristics of the included studies

Study	Design	Country	Sample size	Age	Sex (male), n	Weight, kg	BMI, kg/m^2^
Kahleova 2018 [45]	RCT	USA	38	52.6 (14.7)	2	/	33.1 (31.8–34.3)
Basciani 2020 [46]	Pilot study	Italy	16	/	/	102.1 (12.36)	36.1 (4.3)
Kahleova 2020 [44]	RCT	USA	122	53 (10)	17	93.6 (13.8)	33.3 (3.8)
Li 2016 [47]	RCT	USA	17	56 (4)	11	88.1 (2.9)	30.7 (0.6)
Burke 2007a [48]	RCT	USA	35	44.37 (8.4)	28	97.7 (11.5)	/
Burke 2007b [48]	RCT	USA	45	45.4 (8.5)	41	93 (16.2)	/
Barnard 2021 [49]	RCT	USA	30	58.3 (8.4)	22	98.4 (13.2)	33.7 (3.4)

### Impact of the vegan diet on metabolic indexes

**Fig. 2:**
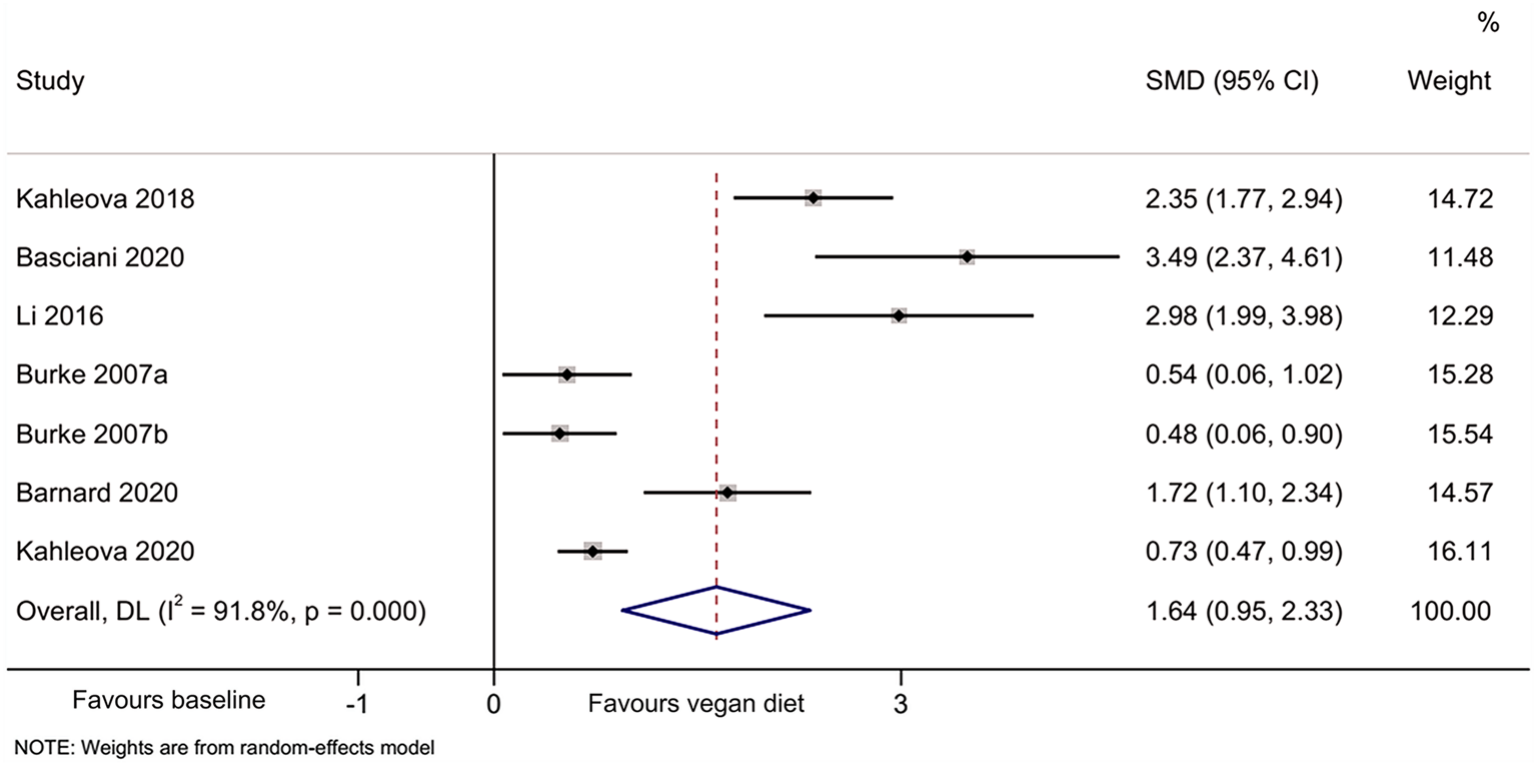
Forest plot illustrating the impact of plant diet on the metabolic parameter of HOMA-IR in obese patients

**Fig. 3:**
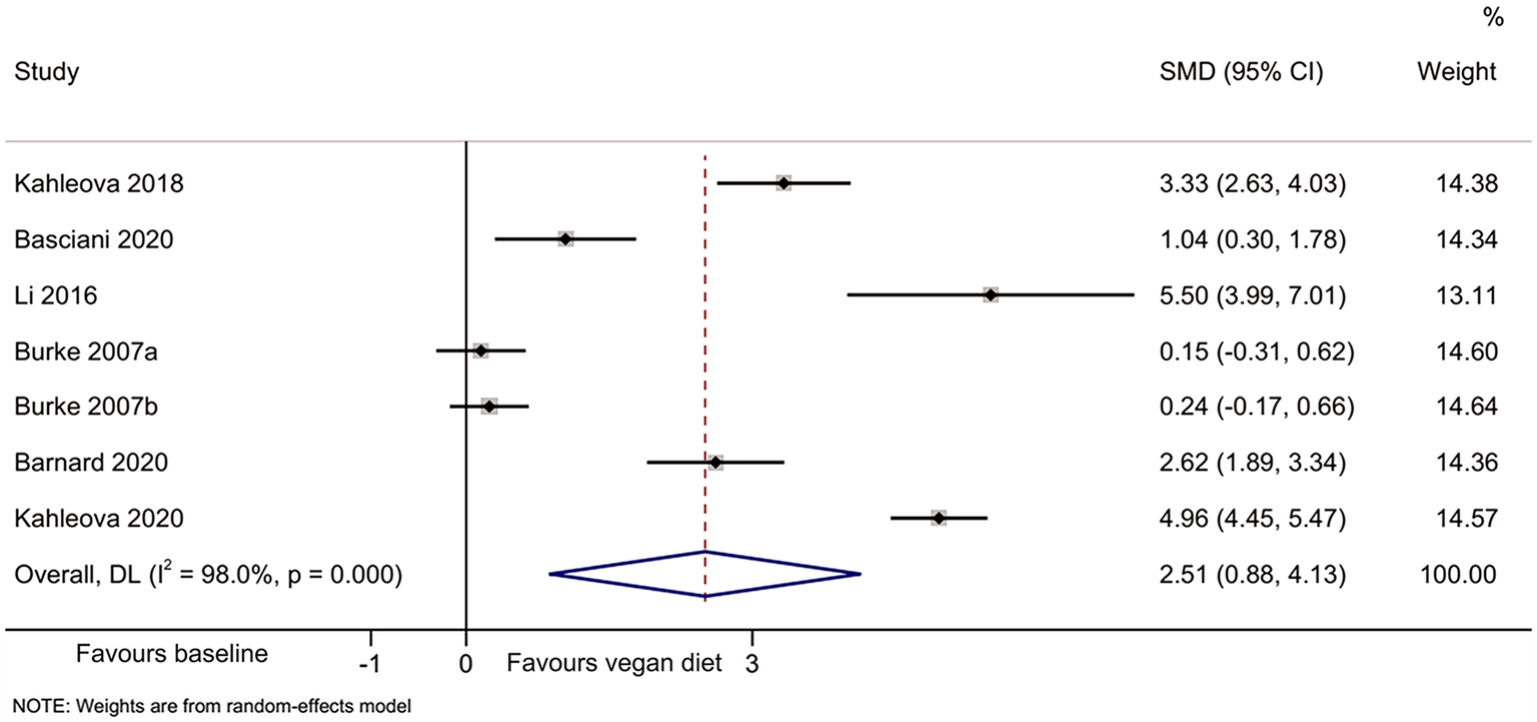
Forest plot illustrating the impact of plant diet on the metabolic parameter of total cholesterol in obese patients

**Fig. 4:**
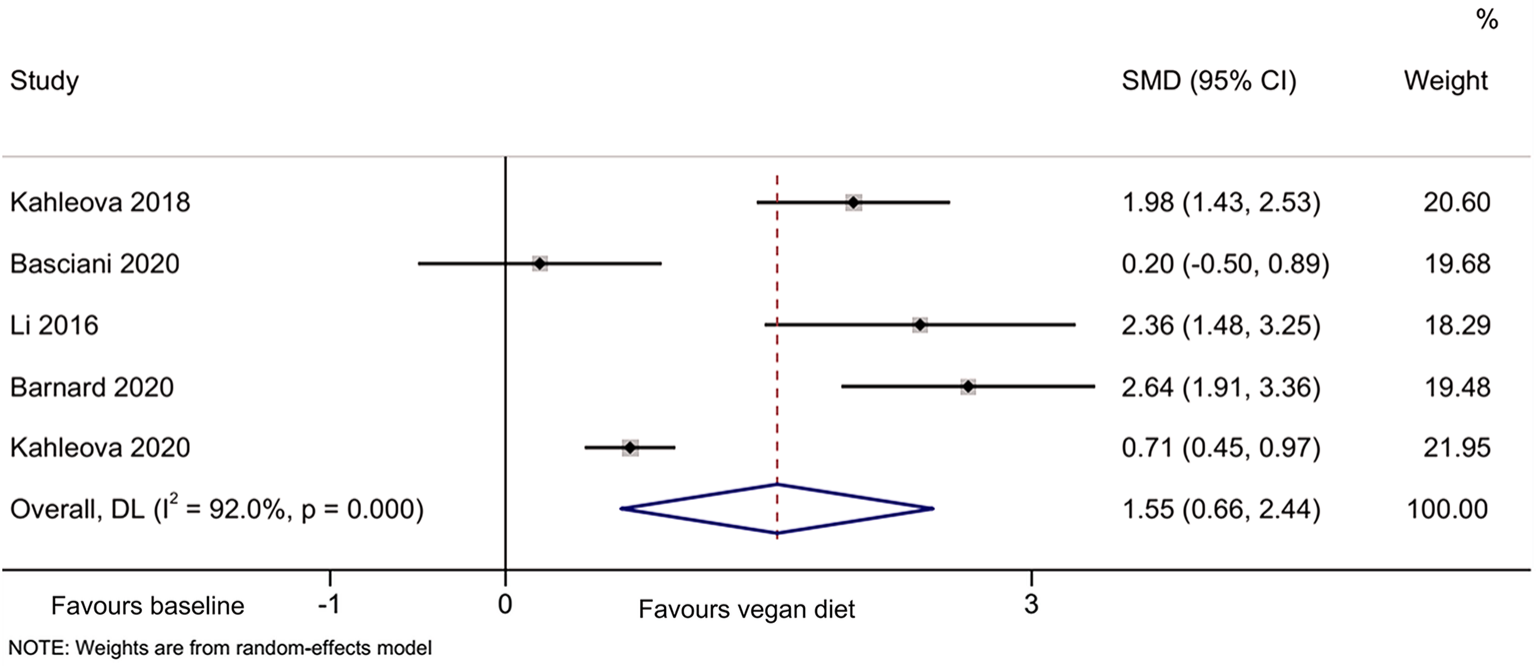
Forest plot illustrating the impact of plant diet on the metabolic parameter of HDL-cholesterol in obese patients

**Fig. 5:**
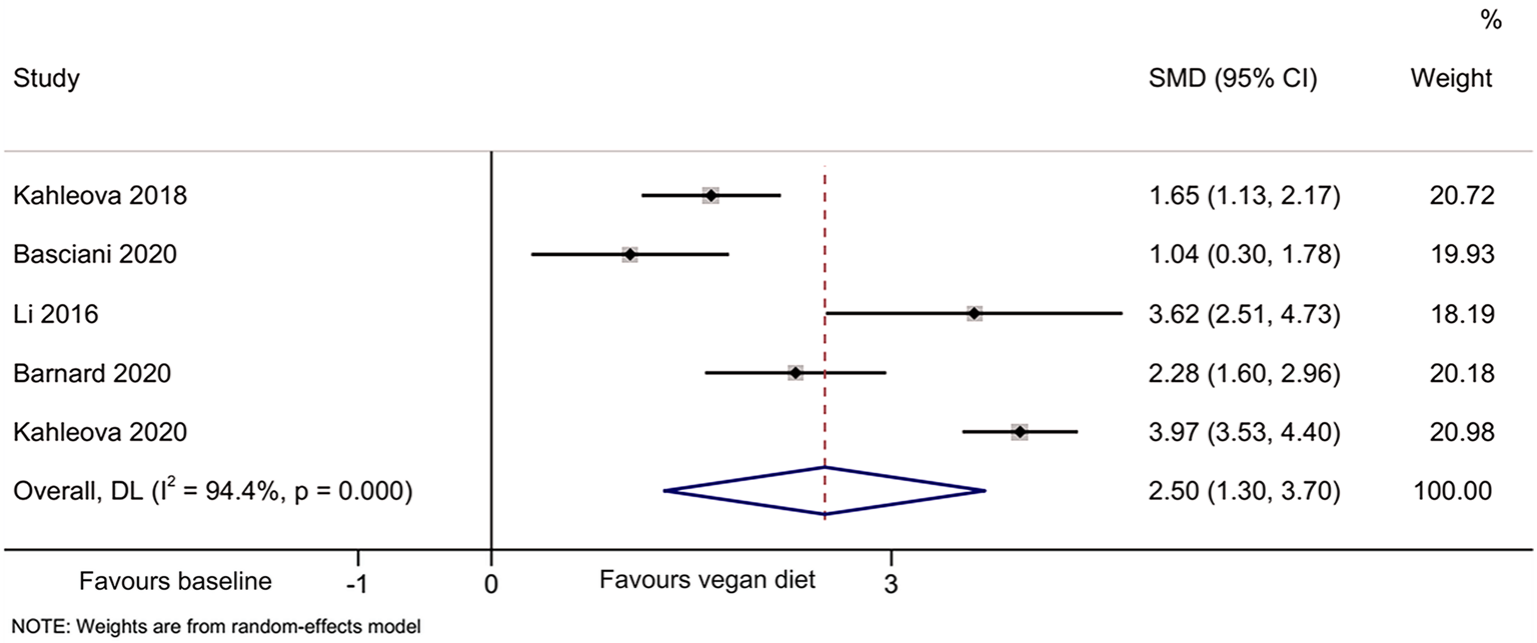
Forest plot illustrating the impact of plant diet on the metabolic parameter of LDL-cholesterol in obese patients

**Fig. 6:**
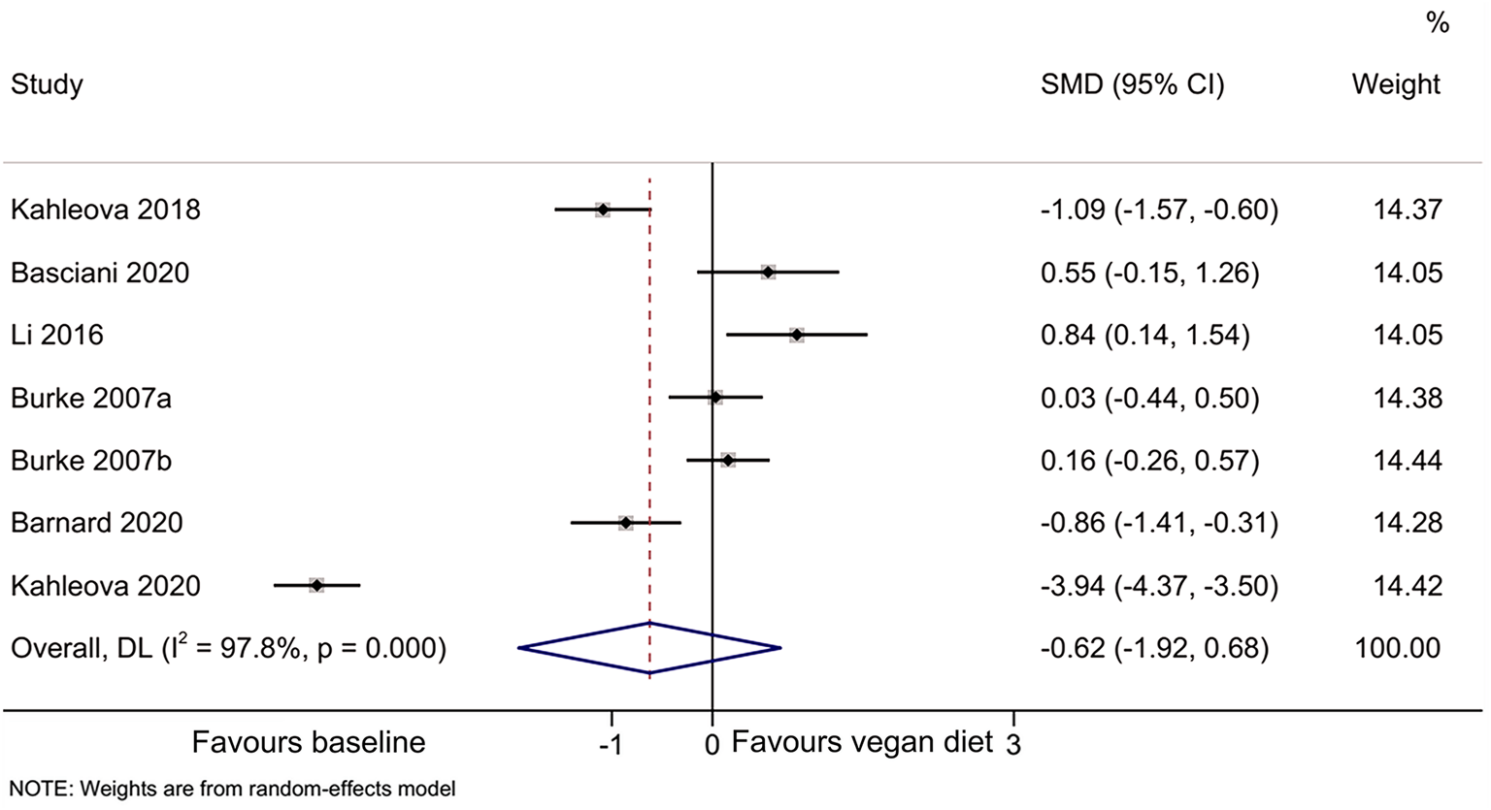
Forest plot illustrating the impact of plant diet on the metabolic parameter of triglycerides in obese patients

The meta-analyses indicated that compared with baseline, the vegan diet improved the HOMA-IR [44–49] (SMD = 1.64, 95% CI 0.95, 2.33; I^2^ = 91.8%, P_heterogeneity_ < 0.001) (Fig. 2), total cholesterol [44–49] (SMD = 2.51, 95%CI: 0.88, 4.13; I^2^ = 98.0%, P_heterogeneity_ < 0.001) (Fig. 3), HDL-cholesterol [44–47, 49] (SMD = 1.55, 95% CI 0.66, 2.44; I^2^ = 92.0%, P_heterogeneity_ < 0.001) (Fig. 4), and LDL-cholesterol [44–47, 49] (SMD = 2.50, 95% CI 1.30, 3.70; I^2^ = 94.4%, P_heterogeneity_ < 0.001) (Fig. 5), but triglycerides showed no significant difference [44–49] (Fig. 6).

### Sensitivity analysis

The Additional file 1: Figs. S1–S6 show that all analyses were robust. The exclusion of each study, in turn, did not change the results.

## Discussion

Studies showed that compared with other weight-loss diets, individuals who follow a vegetarian or vegan diet are more satisfied and are more likely to adhere to it [25–27]. RCTs showed the metabolic benefits of a vegetarian or vegan diet [28–30]. This meta-analysis aimed to investigate the efficacy of plant-based diets on the metabolic parameters of patients with obesity and insulin resistance. The results indicate that in obese individuals with insulin resistance, a vegan diet improves insulin resistance and dyslipidemia, except for triglycerides. Whether these changes result in changes in morbidity and mortality remains to be examined.

The present meta-analysis showed that a vegan diet improved the HOMA-IR in obese individuals with insulin resistance. It is supported by a previous meta-analysis that showed that a vegetarian diet could prevent the development of T2DM [32]. Such a relationship is independent of BMI [50–52]. Vegans also have low levels of intramyocellular lipids related to improved insulin sensitivity [53]. In addition, low consumption of saturated lipids [54, 55] and low liver fat content [56] participate in a better β-cell function. The effect of vegetarian and vegan diets on insulin resistance has been documented by other studies [36, 57–60].

The present meta-analysis showed that the vegan diet improved the blood cholesterol parameters but not the triglycerides. Similar results were reported by a previous meta-analysis [37]. Still, other studies reported conflicting results. Some studies showed that a vegetarian diet improved cholesterol and triglycerides [61, 62], while others reported changes in cholesterol but not HDL-C and triglycerides [63, 64]. A meta-analysis showed that vegetarian diets improved HDL-C [65], and another showed improvement in triglycerides [66]. Still, the changes could depend upon obesity and leptin levels [67, 68], which could explain the conflicting results, at least in part. Nevertheless, all studies agree that a vegetarian or vegan diet induces some beneficial changes in blood lipids.

Substantial heterogeneity was observed in all analyses. Even if all studies examined a vegan diet, there were some differences among the studies, including the exact composition of the diet and the caloric target. Of note, the definition of a vegetarian diet varies in the literature, but the definition of a vegan diet is the same [69–72]. The proportion of males varied from 5 to 91%, and it is well known that obesity, glucose metabolism, and blood lipids display differences between men and women [73]. Future studies should examine the sex differences or be specific to one sex. In addition, the methods to compensate for nutrient deficiencies varied among studies and can influence the results of glucose and lipid metabolism.

Of course, a meta-analysis is always limited by the limitations of each included study, and caution must be applied while extrapolating our results. As for any diet study, the self-reporting of dietary intake has well-known limitations. It is impossible to eliminate uncertainty regarding participants’ adherence. Nevertheless, the studies showed that the reported diet changes were accompanied by changes in weight and plasma lipid levels, suggesting reasonable adherence.

## Conclusions

In conclusion, in obese individuals with insulin resistance, a vegan diet improves insulin resistance and dyslipidemia, except for triglycerides.

## Supplementary Information

**Additional file 1: Figure S1.** Sensitivity analysis of HOMA-IR. **Figure S2.** Sensitivity analysis of total cholesterol. **Figure S3.** Sensitivity analysis of HDL-cholesterol. **Figure S4.** Sensitivity analysis of LDL-cholesterol. **Figure S5.** Sensitivity analysis of triglycerides. **Figure S6.** Sensitivity analysis to test the robustness of the effectiveness of a plant diet on the metabolic parameter triglycerides by sequentially excluding individual studies. **Table S1.** Search processes in PubMed, Embase, and the Cochrane Library.

## Data Availability

The datasets used and/or analyzed during the current study are available from the corresponding author on reasonable request.
