# Supplementary material for: A vegan diet improves insulin resistance in individuals with obesity: a systematic review and meta-analysis
Source: Diabetol Metab Syndr. 2022 Aug 13;14:114. doi: 10.1186/s13098-022-00879-w (PMC9375406; doi:10.1186/s13098-022-00879-w)
Supplement: Supplementary file 1 — Additional file 1: Figure S1. Sensitivity analysis of HOMA-IR. Figure S2. Sensitivity analysis of total cholesterol. Figure S3. Sensitivity analysis of HDL-cholesterol. Figure S4. Sensitivity analysis of LDL-cholesterol. Figure S5. Sensitivity analysis of triglycerides. Figure S6. Sensitivity analysis to test the robustness of the effectiveness of a plant diet on the metabolic parameter triglycerides by sequentially excluding individual studies. Table S1. Search processes in PubMed, Embase, and the Cochrane Library. [file 13098_2022_879_MOESM1_ESM.docx]

**A vegan diet improves insulin resistance in individuals with** **obesity: A systematic review and meta-analysis**

**Running title:** Vegan diet and metabolic indexes

Peng Chen^1^, Ying Zhao^2^, Yan Chen^2*^

^1^Department of Pediatrics, The Second Hospital of Jilin University, Changchun, China

^2^Department of Endocrinology, The Second Hospital of Jilin University, Changchun, China

***Corresponding author:**

Yan Chen

Department of Endocrinology, The Second Hospital of Jilin University

No. 218, Ziqiang Street, Nanguan District, Changchun, China

Tel: 86-043181136436

E-mail: cheny99@jlu.edu.cn

**Supplementary Figures**

**
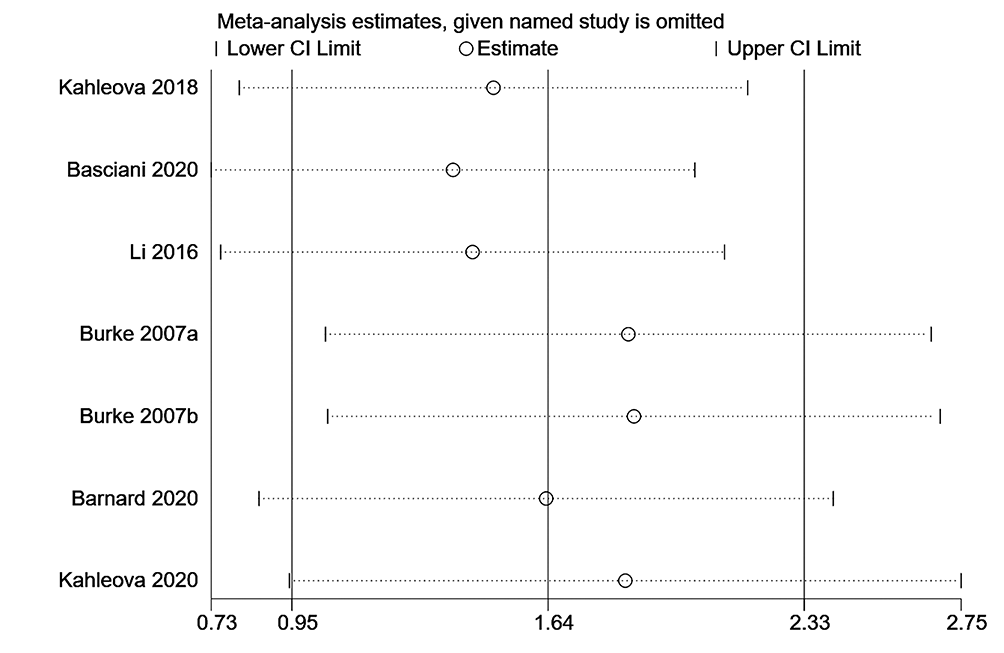
**

**Figure S1.** Sensitivity analysis of HOMA-IR

**
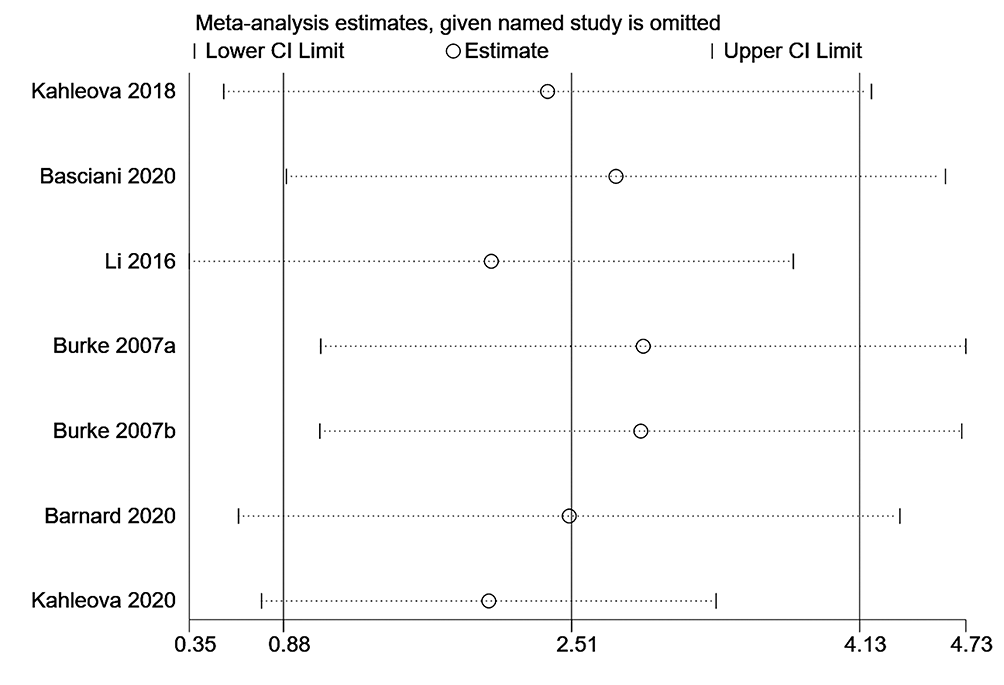
**

**Figure S2.** Forest plot of total cholesterol

**
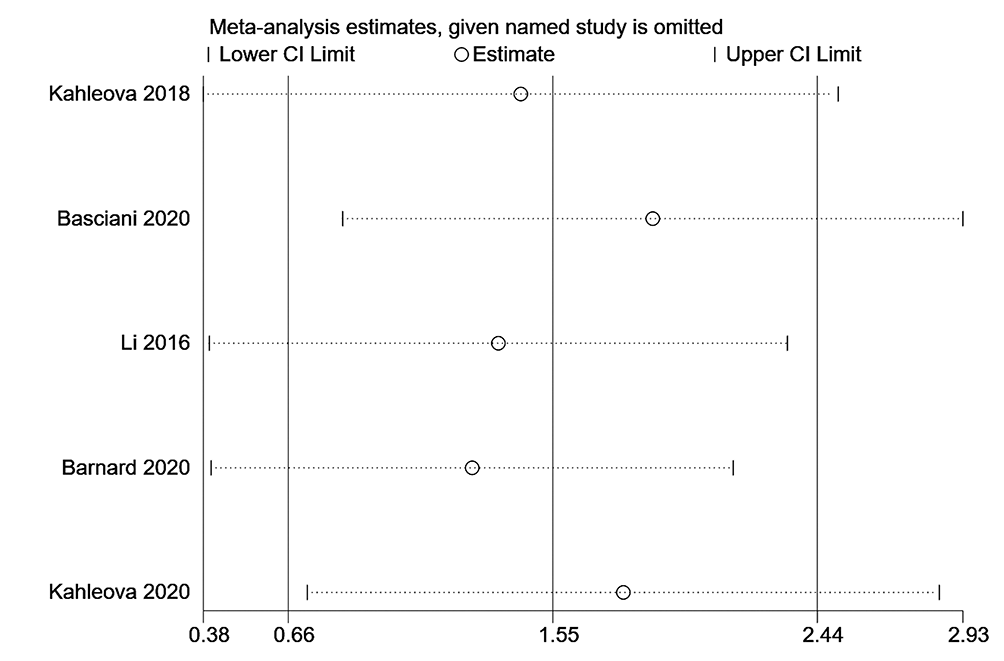
**

**Figure S3.** Forest plot of HDL-cholesterol

**
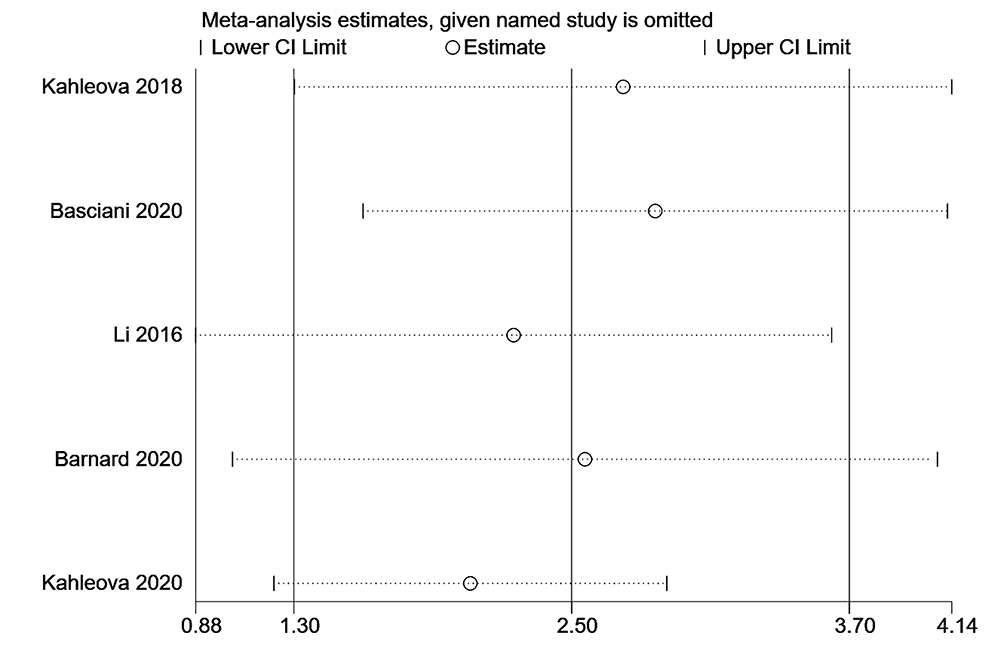
**

**Figure S4.** Forest plot of LDL-cholesterol

**
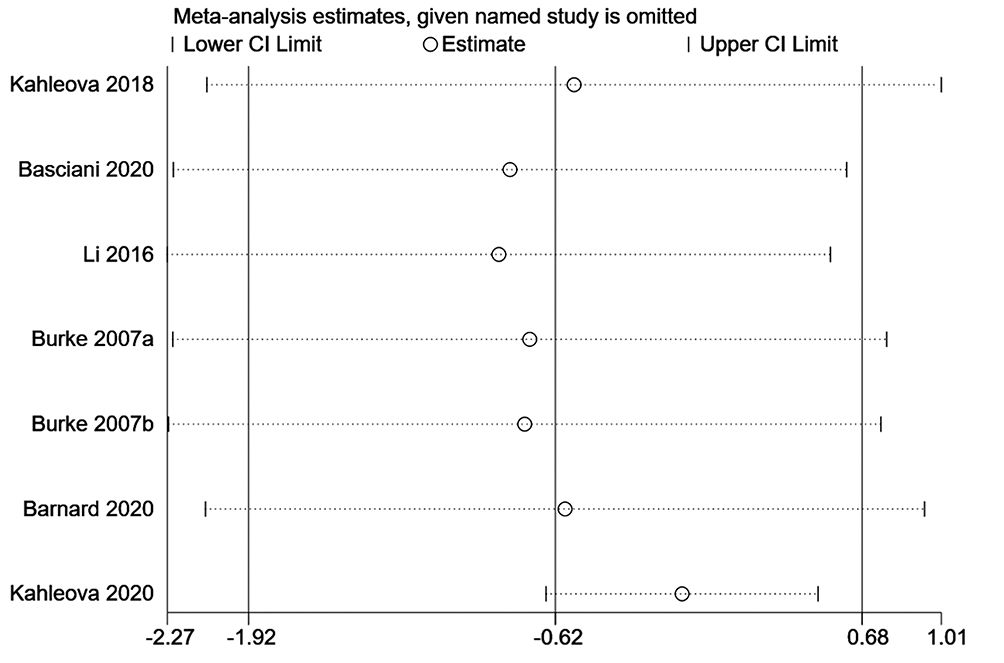
**

**Figure S5.** Forest plot of triglycerides


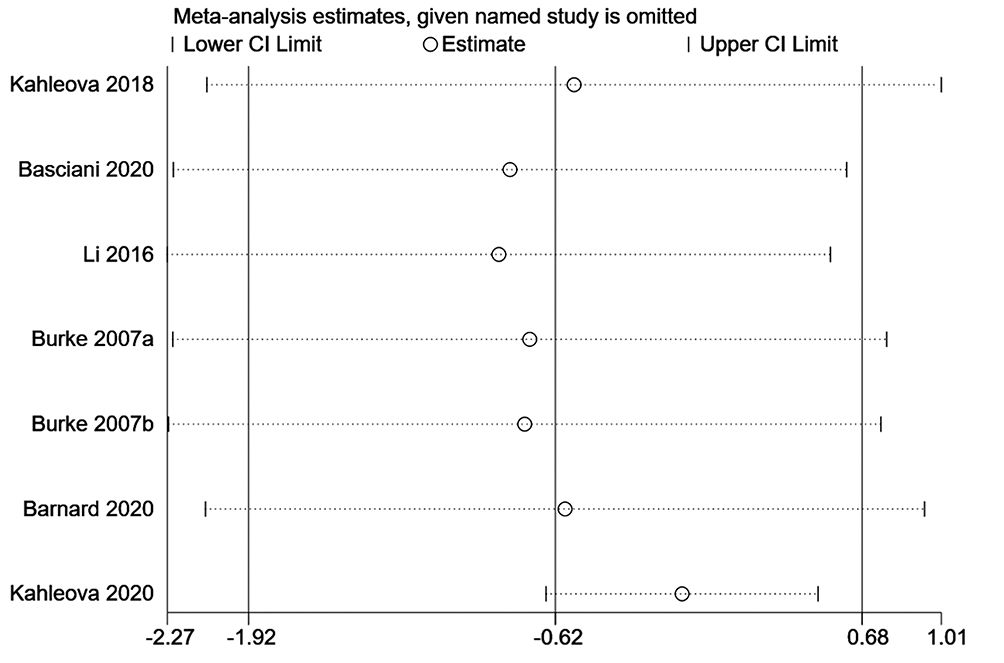


**Figure S6.** Sensitivity analysis to test the robustness of the effectiveness of a plant diet on the metabolic parameter triglycerides by sequentially excluding individual studies.

**Supplementary Tables**

**Supplementary Table S1.** Search processes in PubMed, Embase, and the Cochrane Library

| **PubMed** |  | **Search strategy** | **Numbers** |
| --- | --- | --- | --- |
| **Patient** | **#1** | Obse* OR Overweight | 3,990,430 |
| **Intervention** | **#2** | Diets, Vegan[TIAB] OR Vegan Diets[TIAB] OR Vegan Diet[TIAB] OR Veganism[TIAB] OR Diets, Vegetarian[TIAB] OR Vegetarian Diets[TIAB] OR Vegetarian Diet[TIAB] OR Lacto-Vegetarian Diet[TIAB] OR Diet, Lacto-Vegetarian[TIAB] OR Diets, Lacto-Vegetarian[TIAB] OR Lacto Vegetarian Diet[TIAB] OR Lacto-Vegetarian Diets[TIAB] OR Plant-Based Diet[TIAB] OR Diets, Plant-Based[TIAB] OR Plant Based Diet[TIAB] OR Plant-Based Diets[TIAB] OR Diet, Plant-Based[TIAB] OR Diet, Plant Based[TIAB] OR Plant-Based Nutrition[TIAB] OR Nutrition, Plant-Based[TIAB] OR Plant Based Nutrition[TIAB] OR Lacto-Ovo Vegetarian Diet[TIAB] OR Diet, Lacto-Ovo Vegetarian[TIAB] OR Diets, Lacto-Ovo Vegetarian[TIAB] OR Lacto Ovo Vegetarian Diet[TIAB] OR Lacto-Ovo Vegetarian Diets[TIAB] OR Vegetarian Diet, Lacto-Ovo[TIAB] OR Vegetarian Diets, Lacto-Ovo[TIAB] OR Vegetarianism[TIAB] OR "Diet, Vegetarian"[Mesh] OR "Diet, Vegan"[Mesh] | 5,038 |
| **Outcome** | **#3** | Resistance, Insulin OR Insulin Sensitivity OR Sensitivity, Insulin | 151,360 |
|  | **#4** | #1 AND #2 AND #3 | 68 |

| Embase |  | Search strategy | Numbers |
| --- | --- | --- | --- |
| Intervention | **#1** | 'diet, vegetarian':ab,ti OR 'plant-based diet':ab,ti OR 'vegetarian eating patterns':ab,ti OR 'vegetarianism':ab,ti OR 'vegetarian diet'/exp | 5213 |
|  | **#2** | 'vegan diet'/exp OR 'diet, vegan':ab,ti OR 'veganism':ab,ti | 693 |
|  | **#3** | #1 OR #2 | 5,228 |
| Patient | **#4** | Obes* OR Overweight | 667,057 |
|  | **#5** | 'insulin resistance'/exp OR 'insulin resistance' | 160,387 |
|  | **#6** | 'insulin sensitivity'/exp OR 'insulin sensitivity' | 64,119 |
|  | **#7** | #5 OR #6 | 189,037 |
| Outcome | **#8** | #3 AND #4 AND #7 | 87 |

| Cochrane |  | Search strategy | Numbers |
| --- | --- | --- | --- |
| Patient | **#1** | MeSH descriptor: [Diet, vegetarian] explode all trees | 218 |
|  | **#2** | (Vegetarian Diet:ti,ab,kw) OR (Vegetarian Diets:ti,ab,kw) OR (Diets, Vegetarian:ti,ab,kw) OR (Diet, Plant Based:ti,ab,kw) OR (Plant-Based Diets:ti,ab,kw) OR (Plant Based Diet:ti,ab,kw) OR (Diets, Plant-Based:ti,ab,kw) OR (Diet, Plant-Based:ti,ab,kw) OR (Plant-Based Diet:ti,ab,kw) OR (Vegetarianism:ti,ab,kw) OR (Lacto-Ovo Vegetarian Diet:ti,ab,kw) OR (Lacto-Ovo Vegetarian Diets:ti,ab,kw) OR (Diet, Lacto-Ovo Vegetarian:ti,ab,kw) OR (Diets, Lacto-Ovo Vegetarian:ti,ab,kw) OR (Vegetarian Diets, Lacto-Ovo:ti,ab,kw) OR (Vegetarian Diet, Lacto-Ovo:ti,ab,kw) OR (Lacto Ovo Vegetarian Diet:ti,ab,kw) OR (Plant-Based Nutrition:ti,ab,kw) OR (Nutrition, Plant-Based:ti,ab,kw) OR (Plant Based Nutrition:ti,ab,kw) OR (Lacto Vegetarian Diet:ti,ab,kw) OR (Lacto-Vegetarian Diet:ti,ab,kw) OR (Diet, Lacto-Vegetarian:ti,ab,kw) OR (Diets, Lacto-Vegetarian:ti,ab,kw) OR (Lacto-Vegetarian Diets) | 1298 |
|  | **#3** | #1 OR #2 | 1472 |
| Intervention | **#4** | MeSH descriptor: [Diet, vegan] explode all trees | 17 |
|  | **#5** | Vegan Diet:ti,ab,kw OR Diets, Vegan:ti,ab,kw OR Vegan Diets:ti,ab,kw OR Veganism | 195 |
|  | **#6** | #4 OR #5 | 195 |
| Outcome | **#7** | #3 OR #6 | 1,369 |
|  | **#8** | MeSH descriptor: [Insulin resistance] explode all trees | 6,412 |
|  | **#9** | Resistance, Insulin OR Insulin Sensitivity OR Sensitivity, Insulin | 19,878 |
|  | **#10** | #8 OR #9 | 21,140 |
|  | **#11** | Obes* OR Overweight | 50,404 |
|  | **#12** | #7 AND #10 AND #11 | 86 (63 trials) |
